# Pachydermoperiostosis in a patient with chronic hepatitis B virus infection referred as acromegaly: a case report

**DOI:** 10.1186/s13256-018-1578-2

**Published:** 2018-03-08

**Authors:** Yacoba Atiase, Ernest Yorke, Josephine Akpalu, Bismark Opoku-Asare, Patrick Adjei, Maame Boatemma Amissah-Arthur, Albert Akpalu

**Affiliations:** 1 Department of Medicine and Therapeutics, School of Medicine and Dentistry, College of Health Sciences, University of Ghana, Legon, Accra, Ghana; 2 Department of Medicine and Therapeutics, Korle Bu Teaching Hospital, Accra, Ghana

**Keywords:** Pachydermoperiostosis, Primary hypertrophic osteoarthropathy, Hepatitis B virus, Acromegaly

## Abstract

**Background:**

Primary hypertrophic osteoarthropathy also known as pachydermoperiostosis is a rare genetic disorder that has often been confused with acromegaly because of similar clinical features. Vascular endothelial growth factors which have been implicated in the clinical features of pachydermoperiostosis, have also been shown to be present in chronic hepatitis and implicated in the malignant transformation of hepatitis B infection to hepatocellular carcinoma. To the best of our knowledge there is one reported case of pachydermoperiostosis with chronic hepatitis B infection. We do not imply a causal relationship between pachydermoperiostosis and hepatitis B infection because pachydermoperiostosis is a genetic disorder; however, the question is raised whether hypertrophic osteoarthropathy is one of the many extrahepatic manifestations of chronic hepatitis B infection.

**Case presentation:**

A 21-year-old African (Ghanaian) man with chronic hepatitis B infection was referred to our Endocrine unit as having acromegaly with changing facial features, enlarging hands and feet, and large knee joint effusions which affected activities of daily living. He was finally diagnosed as having pachydermoperiostosis when acromegaly, rheumatological disorders, as well as cardiopulmonary disorders were ruled out. He improved with arthrocentesis, a tapering regime of steroids, non-steroidal anti-inflammatory drugs, and proton pump inhibitors.

**Conclusions:**

The possible role of hepatitis B in hypertrophic osteoarthropathy, that is, secondary hypertrophic osteoarthropathy, needs to be explored; however, with digital clubbing in his father our patient is likely to have pachydermoperiostosis.

## Background

Hypertrophic osteoarthropathy may be primary or secondary. Primary hypertrophic osteoarthropathy is a rare genetic disorder transmitted commonly in an autosomal dominant pattern, but autosomal recessive and X-linked transmission have been described [1]. Specific clinical features include digital clubbing, pachydermia, periostosis, and enlargement of hands and feet due to periarticular and osseous expansion. Pachydermoperiostosis (PDP), often confused with acromegaly based on the clinical features [2, 3], typically occurs in adolescent males and people of African ancestry.

The secondary form is usually a paraneoplastic syndrome that is commonly seen with pulmonary diseases such as lung cancer [4] with predominantly bone changes and fewer dermatological features.

We are not certain of the mechanisms underlying PDP, but there is some suggestion that fibroblast activation [5], vascular endothelial growth factor (VEGF), and platelet-derived growth factor (PDGF) may have a role [6, 7]. Recently a case of PDP with chronic hepatitis B infection was published [8]. It has been suggested that the virus may not be the sole causative agent for malignant transformation in hepatitis B infection but angiogenic factors similar to those in PDP play a role [9–11]; hepatitis B is associated with many extrahepatic manifestations but it is not known whether hypertrophic osteoarthropathy is one of them, this needs further investigation and exploration.

We present a case of PDP with chronic hepatitis B infection that was referred to our Endocrine clinic as a case of acromegaly.

## Case presentation

### Patient information

A 21-year-old Ghanaian man presented initially with a 6-year history of progressively worsening pain and swelling in both knee and wrist joints, which moderately affected his activities of daily living. He reported episodes of fever and chills in the past, although these were absent at the time of presentation. He had polyuria, polydipsia, and nocturia but he did not have weight loss, headaches, or loss of vision.

He had profuse diaphoresis particularly of his face, hands, and feet but did not have any other symptoms of hyperthyroidism. He had noticed an increase in the size of his hands and feet and a change in his facial appearance which were his main concerns. He had been treated with analgesics in the past, which only transiently relieved his pain.

There was no past medical history of diabetes or sickle cell disease. He had been diagnosed as having chronic hepatitis B infection 4 years prior to seeing us but was not on any treatment. There is no family history of diabetes, sickle cell disease, or a presentation similar to his.

He is the second of three children of his parents who are both alive; no sibling has any stigmata of PDP. His father has three other children with another woman who have no stigmata of PDP. His mother has one surviving sibling with four children of whom none have stigmata of PDP. His other maternal cousins also do not have any stigmata of PDP.

His illness has taken a psychological toll on him because he has been unable to continue his education after secondary school although he excelled in his examinations and got a scholarship to the university. He is usually at home indoors, because of people’s comments about his appearance when he goes out. His older sister has had two suitors renege on their promise to marry her on meeting him for fear the disorder could be familial and be transmitted to their offspring.

### Clinical findings

**Fig. 1:**
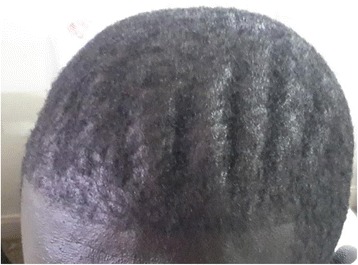
Cutis verticis gyrata

**Fig. 2:**
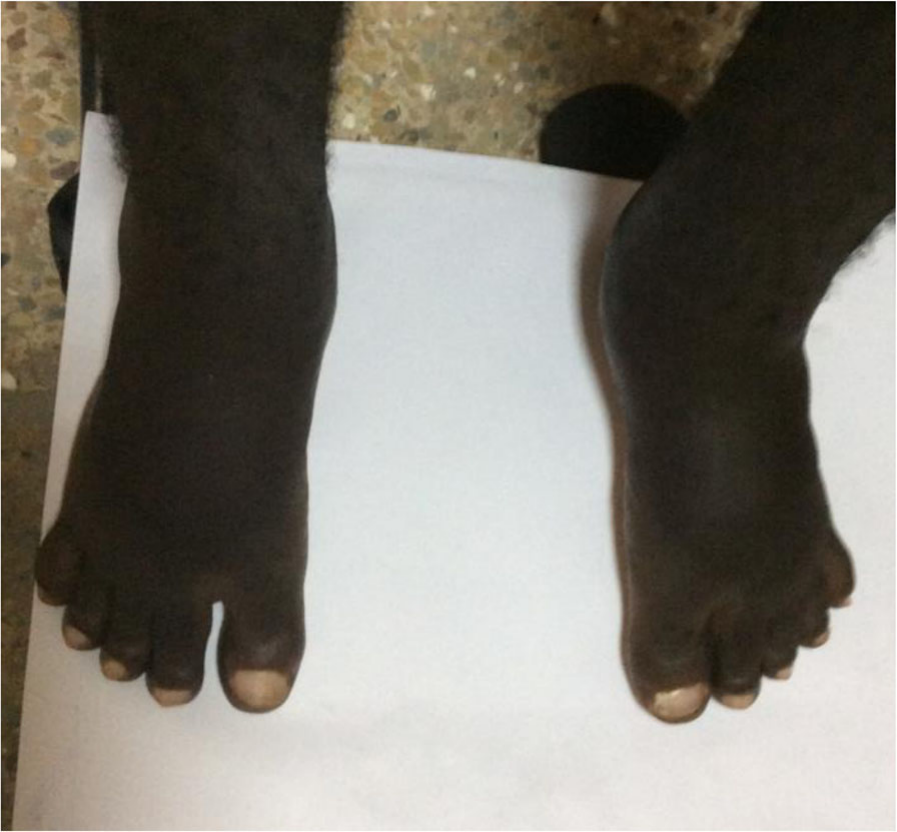
Spade-like feet and clubbing of toes

**Fig. 3:**
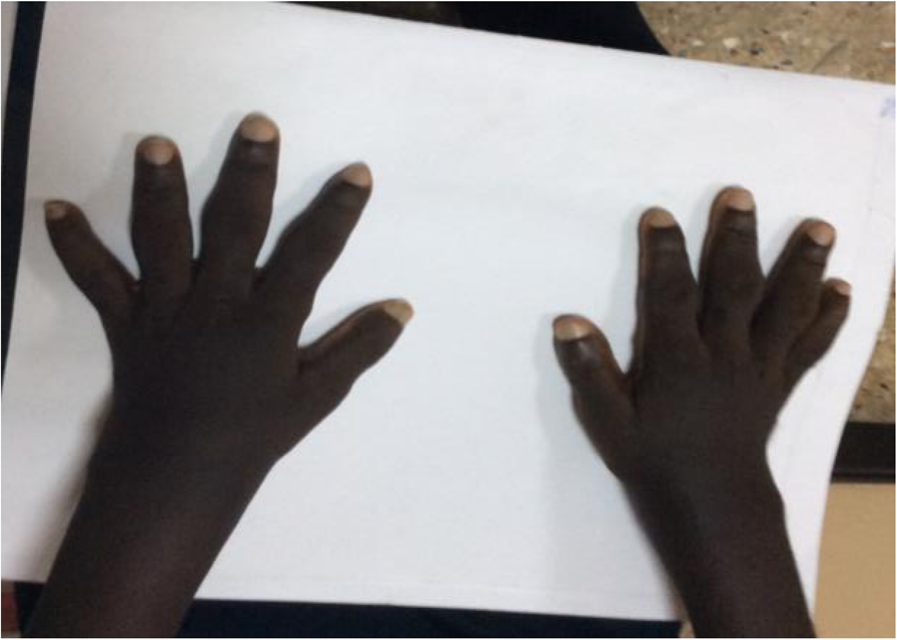
Clubbing of fingers

An examination revealed a young man with coarse facial features, hyperhidrosis, cutis verticis gyrata (Fig. 1), pectus excavatum, doughy palms, spade like hands and feet (Fig. 2), digital clubbing (Fig. 3), and pitting pedal edema at his ankles with profusely diaphoretic hands and feet. There was no cyanosis, jaundice, skin rash, or any stigmata of chronic liver disease. He had visual field defects in the superior temporal quadrant bilaterally on confrontation, mild wasting and weakness of proximal muscles, and an antalgic gait. His cardiorespiratory and abdominal examinations were normal.

**Fig. 4:**
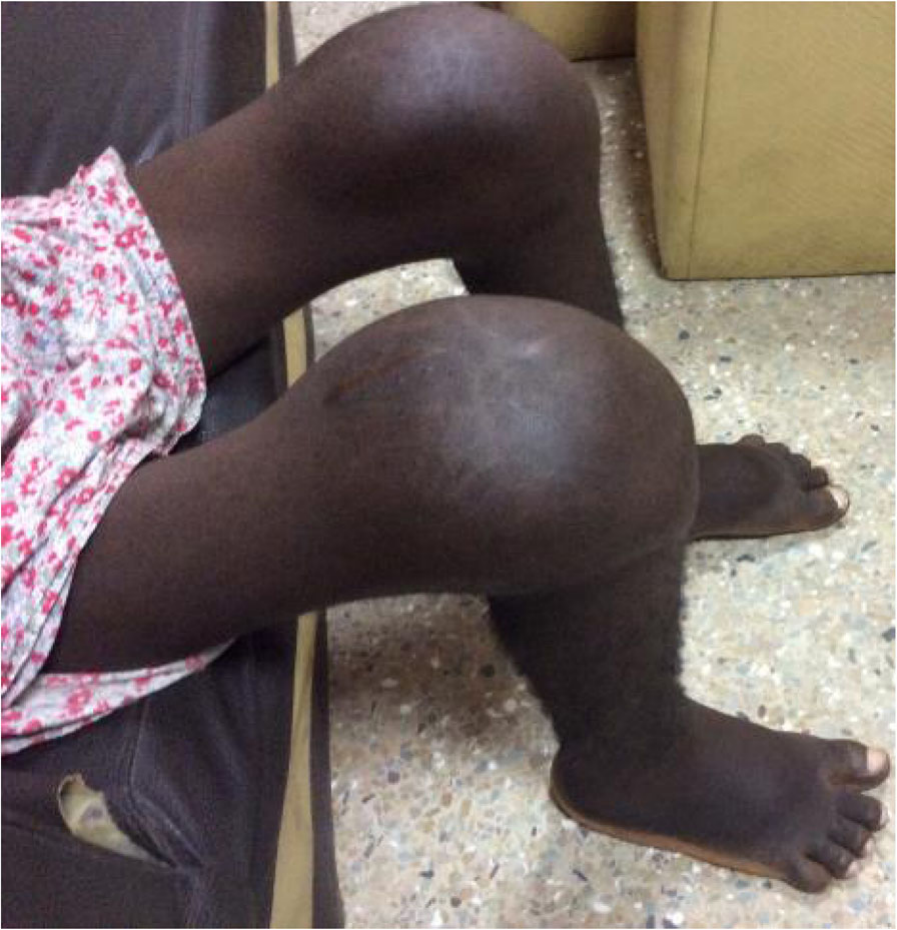
Massive bilateral knee joint effusions, with striae of overlying skin and clubbing of toes

Significant musculoskeletal findings included massive effusions of both knees with striae of the overlying skin (Fig. 4) associated with limited range of movement. There was also evidence of bone expansion at his wrist joints without soft tissue swelling, tenderness, or warmth. There was reduced flexion and extension as well as crepitus at his wrist joints. He also had enlarged proximal interphalangeal (PIP) joints bilaterally with a good handgrip.

### Diagnostic assessment

Laboratory tests revealed mild normocytic normochromic anemia with hemoglobin of 10.9 g/dl. However, the following tests were within normal limits: erythrocyte sedimentation rate (ESR), fasting plasma glucose at 5.0 mmol/l, serum insulin-like growth factor 1 (IGF1), liver, renal, thyroid function tests, and serum corrected calcium of 2.32 mmol/L. His serum albumin level was 33 g/L (35–50 g/L). Preliminary investigations for a rheumatological condition such as rheumatoid factor, anticyclic citrullinated peptide (anti-CCP), antinuclear antibody (ANF), and creatinine kinase (CK) were all normal. A chest X-ray, echocardiogram, abdominal ultrasound, pelvic ultrasound, and magnetic resonance imaging (MRI) of his brain were also normal with no pituitary lesion seen; these findings ruled out rheumatological diseases, cardiopulmonary diseases, or acromegaly from a pituitary adenoma as a cause of the clinical features. He has financial constraints as the family is unwilling to support him because he refused to seek alternative treatment.

### Therapeutic interventions

Arthrocentesis done under aseptic conditions yielded approximately 700 ml of straw colored, normal viscosity aspirate per knee joint. There was residual effusion after this therapeutic and diagnostic aspiration. Gram stain, culture, cytology, cell count, and analysis for crystals were normal with no bacterial growth.

**Fig. 5:**
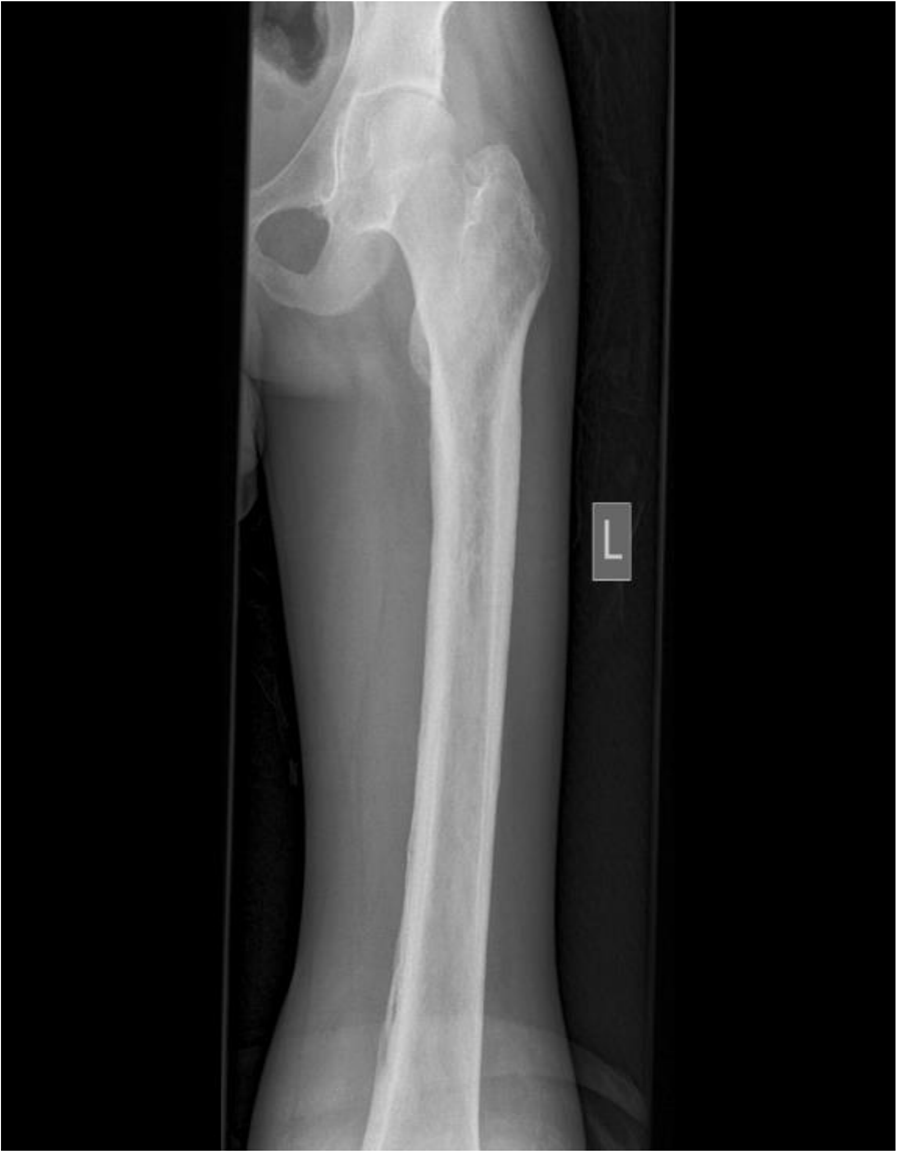
X-ray of femur showing periosteal thickening of medial cortex of femur

**Fig. 6:**
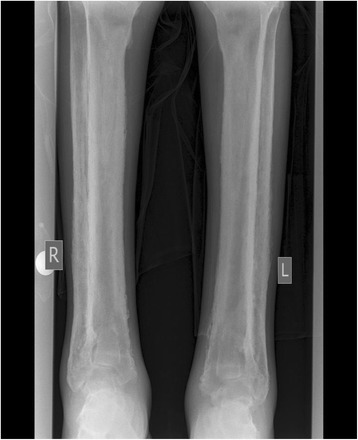
X-rays of tibia and fibula showing periosteal thickening

**Fig. 7:**
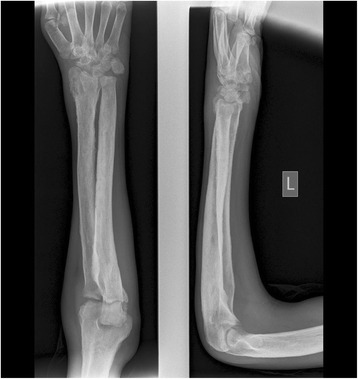
X-rays of ulna and radius showing periosteal thickening of both ulnae and medial radial shafts

**Fig. 8:**
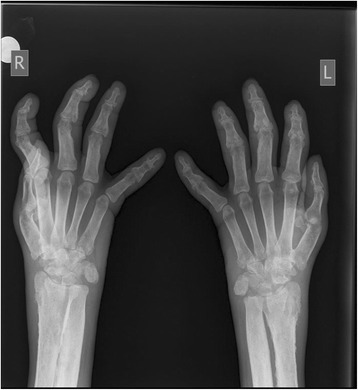
X-rays of hands showing expansion of ulna shaft and thickening of second to fourth digits

X-rays of his lower limbs showed periosteal thickening of the medial cortices of both femurs with sparing of the lateral cortices (Fig. 5). There was uninterrupted thickening of the periosteum of both lateral and medial cortices of his tibia and fibula (Fig. 6). Effusions of both knee joints were noted. The X-rays of his wrist joints, radius, and ulna showed diffuse bilateral symmetric periosteal thickening with marginal irregularities of both ulnae and medial aspects of both radial shafts (Fig. 7). There was expansion of the ulna shafts with flaring of both distal radii and a suggestion of cortical thickening in the ulna aspects of the second to fourth digits of both hands (Fig. 8).

The effusion accumulated rapidly within a week. He was initiated on prednisolone 40 mg daily, which was reduced by 10 mg per week over 6 weeks. He was also given diclofenac 75 mg twice a day and omeprazole 20 mg twice a day over a month. Physiotherapy was started with active and active-assisted exercises of both upper and lower limbs. A bone biopsy was considered but was not done; bone scans or genetic testing were not done either. Three weeks after admission, he was discharged on prednisolone 10 mg which he took until week 4 and was weaned off by week 6. His pain improved but did not resolve completely and he was walking unaided and performing tasks of daily living better than when he was admitted. He is currently on celecoxib 100–200 mg *pro re nata* (PRN; as needed) and physiotherapy.

### Follow up and outcomes

**Fig. 9:**
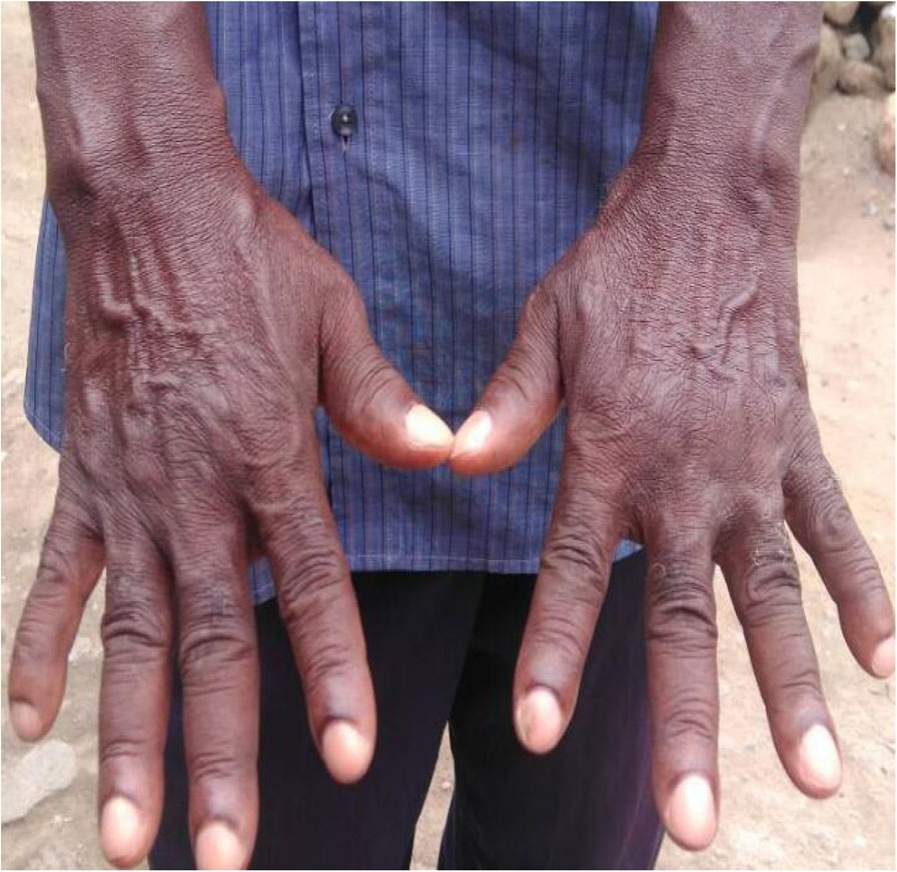
Father’s hands showing digital clubbing

**Fig. 10:**
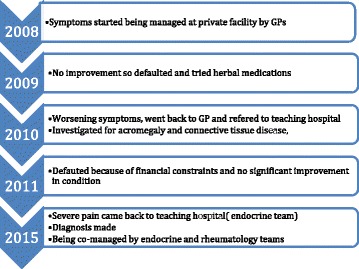
Time course of illness

At a review, 3 weeks after discharge, with his father, we noticed his father had clubbing of his fingers (Fig. 9). There were no other symptoms and signs of pulmonary disease or hypertrophic osteoarthropathy in his father. Genetic testing was considered then but this was not available in our hospital and our patient’s father declined the offer to screen him for cardiopulmonary disease with X-rays. The time course of our patient’s illness is shown in Fig. 10.

## Discussion

In 1991, a diagnostic criteria for PDP was proposed [12] comprising three major criteria namely finger clubbing, periostosis, and pachydermia and nine minor criteria: seborrhea, folliculitis, hyperhidrosis, arthritis or arthralgia, acro-osteolysis, gastric ulcer or gastritis, neurovegetative syndrome (flushing and blanching), hypertrophic gastropathy, and cutis verticis gyrata. For simplicity, three forms have subsequently been described: the “complete form,” the “incomplete form,” and the “forme fruste” [13]. The “complete form” shows three major and some minor criteria, the incomplete form has two major and some minor criteria, and the forme fruste has one major and some minor criteria.

As per these criteria our patient’s diagnosis is a complete form of PDP with three major (finger clubbing, periostosis, and pachydermia) and four minor criteria (seborrhea, hyperhidrosis, arthritis and arthralgia, and cutis verticis gyrata). In addition, the normal biochemical results, radiological findings, echocardiographic findings, and negative screening tests rule out the diagnoses of acromegaly, rheumatological conditions, and cardiopulmonary conditions.

Cutis verticis gyrata, a rarer feature, affects approximately 24% of patients with PDP [14]; cutis verticis gyrata can be seen in acromegaly together with spade-like hands and feet [15]. Clubbing which is found in 89% of patients with PDP [1] may also be found in acromegaly. Seborrhea and hyperhidrosis are clinical features of both PDP and acromegaly and commonly found in adolescence as well. Rarely, acromegaly may be associated with PDP [2].

Neurological complications due to compression of the spinal cord have been mentioned [3, 4] in patients with PDP and may have been responsible for the muscle wasting and weakness found in our patient; this was not investigated in our patient. Anemia found in patients with PDP is often multifactorial and may be due to myelofibrosis, extramedullary hematopoiesis, and gastrointestinal bleeding occurring as a result of peptic ulcer or erosion [5]. These gastric or duodenal ulcers or erosions may be severe and occur in almost 50% of patients [6].

Management includes relieving symptoms of arthritis with steroids and non-steroidal anti-inflammatory drugs (NSAIDs) as was done for our patient who had significant pain at diagnosis. Most patients are said to have mild to moderate discomfort but our patient had significant pain affecting activities of daily living. Colchicine has been shown to improve the articular symptoms, folliculitis, and pachydermia by possibly inhibiting neutrophil chemotaxis and subsequent tissue edema [7]. Skin improves with retinoid treatment, but plastic surgery and botulin toxin have been useful for facial correction which is a major concern in most patients [8]. Genetic counselling and testing should ideally be offered to the family and where this is not possible, as in our patient, a radiologic survey of immediate family members may be useful [9].

Hepatitis B infection is frequently complicated by hepatocellular carcinoma. The virus alone is probably not the sole causative factor in the malignant transformation; angiogenic factors including VEGF have been implicated [9–11]. Of interest, these angiogenic factors including fibroblast activation factor [10], VEGF, and PDGF have also been shown to possibly play a role in PDP [11, 12]. A case of hepatitis B infection and PDP similar to ours has recently been reported [13]. What is not documented to the best of our knowledge is whether VEGF in chronic hepatitis B may play a role in PDP. We are not implying that the relationship between hepatitis B and PDP is causal; however, with a common pathophysiologic link being VEGF and one documented case of PDP and chronic hepatitis, the link between these two conditions may need exploring.

Although we were unable to perform any genetic studies in this patient and his immediate family members, it is very likely that the chronic hepatitis B in our patient was an incidental finding and his was a case of PDP; this was suggested by obvious digital clubbing in his father. It is important to remember that PDP remains largely a clinical diagnosis, confirmed by radiology of the long bones.

## Conclusions

PDP is often investigated as acromegaly and is ruled out by normal biochemical and radiological investigations. We have suggested exploring the link between PDP and chronic hepatitis infection; however, with this patient’s father having digital clubbing we think this case is likely to be primary hypertrophic osteoarthropathy and not secondary hypertrophic osteoarthropathy from chronic hepatitis B infection.

### Patient’s perspective

Our patient admits to feeling depressed occasionally because of the change in his physical appearance and because his family feels his condition is self-inflicted, as he has refused to seek alternative treatment. He has lost friends and has been unable to pursue his dream of being an accountant.

## Abbreviations

ANF: Antinuclear antibody
Anti-CCP: Anticyclic citrullinated peptide;
CK: Creatinine kinase
ESR: Erythrocyte sedimentation rate
IGF1: Insulin-like growth factor 1
MRI: Magnetic resonance imaging
NSAIDs: Non-steroidal anti-inflammatory drugs
PDGF: Platelet-derived growth factor
PDP: Pachydermoperiostosis
PIP: Proximal interphalangeal
PRN: *pro re nata*
VEGF: Vascular endothelial growth factor
